# Toward the Biophilic Residential Regeneration for the Green New Deal

**DOI:** 10.3390/ijerph18052523

**Published:** 2021-03-04

**Authors:** Eun Ji Lee, Sung Jun Park

**Affiliations:** 1 Department of Architecture, Keimyung University, Daegu 42601, Korea; yej@stu.kmu.ac.kr; 2 Department of Architectural Engineering, Keimyung University, Daegu 42601, Korea

**Keywords:** Green New Deal, climate change, biophilia, biophilic design, residential regeneration, sustainable resilience

## Abstract

As climate changes and species extinction accelerate, the global community focuses on Green New Deal plans to promote economic development based on environmental sustainability. The Green New Deal should encourage sustainable resilience in the environment and strengthen the community’s innate ties with natural resources and biodiversity. This study describes biophilic design for sustainable and resilient residential regeneration from the perspective of the Green New Deal, and suggests potential possibilities for these approaches on a residential regeneration scale. A case study clarifies the applicable features of biophilic design in various fields, such as architectural planning and design, technology, and services, and is subdivided according to the scale of residential regeneration (unit, building, and complex). The results of this study suggest new values for existing Green New Deal policies and contribute to the segmentation of residential regeneration projects and the expansion of related industries.

## 1. Introduction

The acceleration of global warming has caused severe climate change that led to environmental pollution, drought, extreme cold and heat waves, and frequent natural disasters, threatening human health and welfare. In particular, along with climate change, the aging and decline of the population in modern society, the economic downturn, and the increase in decrepit housing are accompanied by an imbalance and qualitative decline in cities around the world [1]. With growing concerns over climate change, efforts to promote economic development based on environmental sustainability have been made around the world, and various proposals for the Green New Deal have been raised internationally [2,3]. The Green New Deal means environmental and human-centered sustainable development and focuses on the eco-friendly transformation of the energy supply and demand system, everyday life, and industrial infrastructure [4,5]. As a result, discussions are actively underway on the regeneration of urban and residential areas, and specific planning measures are required to integrate the welfare and environmental protection of local residents and economic growth.

The recent residential regeneration project presents a vision that considers the direction of economic regeneration, focusing on green remodeling, the application of green technology, and the new and renewable-energy industry. However, it also focuses on how to minimize human environmental impact, without considering various social issues such as the aging population and the quality of life of residents. Therefore, for existing environmental regeneration businesses to become more sustainable and resilient, it is important to secure neighborhoods and communities through the restoration of human-nature relations.

Humans evolved in response to the stimulus of the environment and were adapted to live in a natural environment. In fact, this is supported by research results and empirical evidence that the natural environment is preferred much more than the urban environment or built environment [6,7,8]. The Biophilia hypothesis conceptualizes it as a human biological trait, based on the premise that the stimulation of the natural environment induces a genetically positive response [9]. Specifically, biophilia cannot be explained by a single instinct, and it can be achieved by learning rules and iterative experiences to cultivate and effectively function biological tendencies [10]. Biophilia is applied to the architectural environment through biophilic design. The ultimate goal of the biophilic design is to restore a healthy relationship between humans and the natural environment, leading humans to a positive natural experience, offsetting negative environmental factors and creating a relaxed psychological state [11]. In the field of architecture, various classification systems have been proposed to apply biophilic design; it is divided into patterns of biophilic design [12], experiences and attributes of biophilic design [13], depending on the perspective of the researcher. Recently, biophilic design has highlighted health effects and economic benefits when they are applied to work, medical, and educational environments, which are discussed with the expectation of not only the quality living environment but also the economic revitalization of the community [14,15]. The design method utilizing the characteristics of the natural environment supports active physical activities and healthy mental states [11,12]; it also contributes to the securing of natural resources and the creation of new green jobs by cultivating various species in the urban area [14,16]. It is also advantageous because it promotes communication with a sentiment of social belonging to form community integration and a positive sense of society [10,17].

This study focuses on the similarities between the goals and directions pursued by the concept of the Green New Deal and biophilia and proposes the application of biophilic design and its value as a strategy for residential regeneration in response to climate change. The detailed goals of this study are as follows:

- First, it presents a theoretical basis for integrating the concepts of the Green New Deal and biophilia for the regeneration of residential areas in response to climate change and resilience;
- Second, it discusses the potential value and need for regeneration of biophilic design-based dwellings in health, economic, and social aspects;
- Third, through literature reviews, it derives the characteristics of the biophilic design considering the Green New Deal and proposes a biophilic residential regeneration strategy based on the scale of application.

This study covers not only the developments of theories that correspond to the purpose of the study, but also other areas and variables that do not fall within the scope of the subject.

**Figure 1:**
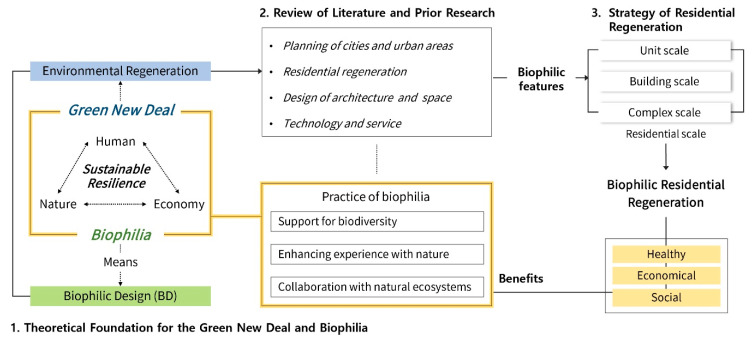
Research scheme.

Figure 1 shows the method and scope to achieve the goals of the study, and the details are as follows. First, it proposes a theoretical basis for the relationship between humans, nature, and the economy and sustainable resilience by considering theories related to the Green New Deal and biophilia concepts. Second, it discusses the health, economic, and social benefits of applying biomedical design by considering prior research related to biophilic design. Third, a case study of biophilic design applications that was conducted in the architectural planning, design, and technology and service industries, which are key areas of the Green New Deal’s environmental regeneration project, is explored. The scope of the survey covers applications of related literature and preceding research, such as urban and residential areas; technologies, including architecture; and service industries, through keyword search. This is to derive a residential regeneration strategy from an integrated and comprehensive perspective. It was conducted using major academic databases, including Scopus, IEEE XPLORE, SpringerLink, ScienceDirect, and PubMed. The details are described in Section 3. Finally, based on the results of the case study, we propose a plan to regenerate the biophilic residential area. The housing regeneration plan proposed in this study is limited to the scope of unit scale, building scale, and complex scale in consideration of the possibility and usability of future applications.

To date, research on environmental regeneration for the Green New Deal has focused on environmental and energy sustainability and community and policy support [18,19,20,21]. However, it is insufficient to consider restoring the relationship between local residents and local ecosystems or to provide support for daily experience with nature. Discussions on biophilic urbanism have been taking place recently, with the emphasis on the impact of the regeneration of urban and residential areas on public health [22,23], but research suggesting a plan for specific applications considering the size and environmental characteristics of a region is lacking. Previous research has also focused on a limited scope, such as the restoration of public facilities and ecosystems, so that it is necessary to discuss economic and technological links, such as Green New Deal projects and smart industries. Amid the rapidly growing demand for the Green New Deal plan, this study discusses the direction of residential regeneration for healthy communities and residents and proposes various applications and the potential value of biophilic design as a key strategy. The results of this study indicate the possibility and need for a link between the Green New Deal project and the biophilic design strategy. Moreover, the results expand the understanding of residential regeneration and the application of biophilic design from the perspective of the Green New Deal. They also contribute the worth of segmenting residential regeneration projects on various scales, the potential utilization of biophilic architecture, and the development of related technologies.

## 2. Green New Deal and Biophilic Design

### 2.1. Climate Change and Green New Deal

Discussions on global weather phenomena and climate change aim at global and national strategic responses to changes in the global environment, including food, soil, energy, and health. Global warming prevents the planet from functioning properly by directly weakening the Earth’s environmental services or disabling them, which inevitably has direct and indirect impacts on various areas, including human life, industrial structure, and the ecosystem [24]. In other words, rapid climate change is expected to pose the most serious threat to the world’s economic, social and environmental stability, requiring continued access to essential goods such as clean water and air, and healthy food and housing.

Climate change is not an independent issue but is closely linked to the growth of the human population, energy, and everyday working and living environments. The fifth evaluation report by the International Panel on Climate Change (IPCC) explains how risks from [25] climate change can adversely affect the human living environment. The impact on the ecosystem may worsen social problems, such as diseases, and resource problems related to human life, such as food scarcity. It is estimated that an increase in average temperature and precipitation will lead to changes in the life cycle, geographical distribution, and species composition of plants and animals. The increase in carbon dioxide levels will have a serious impact on the marine ecosystem; the increase in temperature will cause natural disasters such as floods due to rising sea levels and erosion, accompanied by human casualties [26]. The impact on ecosystems soon leads to social and economic impacts, such as the decline in overall food production due to problems with water supply and demand and the change in the structure of energy supply due to the surge in demand for heating and cooling, increasing the emissions of secondary air pollutants. The system of such a vicious circle is also expected to be prominent in accelerating population migration, with flooding to high-altitude inland areas and an influx of the rural population into cities, which could lead to an imbalance in land use and food supply and demand [27]. Finally, the most serious threat of climate change is health problems, as they create favorable conditions for the spread of diseases, especially among the socially vulnerable. This will deal a heavy blow not only to developing countries but also to all cities suffering from aging populations worldwide. In this context, the living zone concentrated in the urban environment of modern society is expected to suffer huge losses. It is, thus, necessary to improve the quality of the environment and support human health and well-being through environmental management plans, policies, and technology to protect the environment from disasters caused by human activities.

Recently, every country has been pushing for a Green New Deal policy to form a win-win relationship that benefits both by integrating economic growth and environmental protection, which have been perceived as conflicting relations [28,29]. The direction of the New Deal policy in the global era is the convergence of the Digital and the Green New Deal [30], emphasizing the establishment of smart infrastructure in response to the Fourth Industrial Revolution and climate change, discussing environmental and economic responses through the development of green environment and green technologies, and job creation [27,29,31]. The fundamental solution to the problem of climate must be accompanied by extensive academic and industrial efforts, and the construction sector needs to first produce specific countermeasures at the minimum scale of the environment closest to our lives. Whereas the Green New Deal is a hot topic of debate, we are fully aware that the socially vulnerable, animal and plant species, and future generations around the world are already seriously damaged [32]. We should respond to this threat quickly.

The Green New Deal has once again called for an integrated and comprehensive perspective on the issue of residence, as the past way of life, which prioritized progress and development, has changed in a way that values today’s quality of life. In particular, attention is being paid to how to “restore “or “replay” so that each function of “people,” “community,” “nature,” and “economy” can be fulfilled efficiently and effectively [33]. Residential regeneration shall practice regional regeneration considering the identity and environment of the region, and infrastructure shall be established for the city and local community to grow continuously. Therefore, this study focuses on exploring approaches and methodologies to “resilient” residential regeneration, in which the development of residences, nature, and communities can circulate organically to cope with climate change.

### 2.2. Biophilia, from the Point of View of the Green New Deal

The Green New Deal is the coordination of social, ecological, and economic systems in response to current or anticipated climate change and its impact [34], in other words, to build a mutual developmental relationship between humans, nature, and the economy. The approach to the Green New Deal is discussed in convergence with the precautionary aspect, encompassing a multidisciplinary analysis, in which the production and use of energy is a combination of natural scientific perspectives [25,35,36,37]. The preventive approach focuses on “safe management rather than regret”, a precautionary principle that is irreversible in the event of losses caused by climate change [25]. This study deals with the regeneration of residential areas for the Green New Deal and discusses the link with the concept of biophilia in consideration of the direction of preventive and convergent approaches.

**Figure 2:**
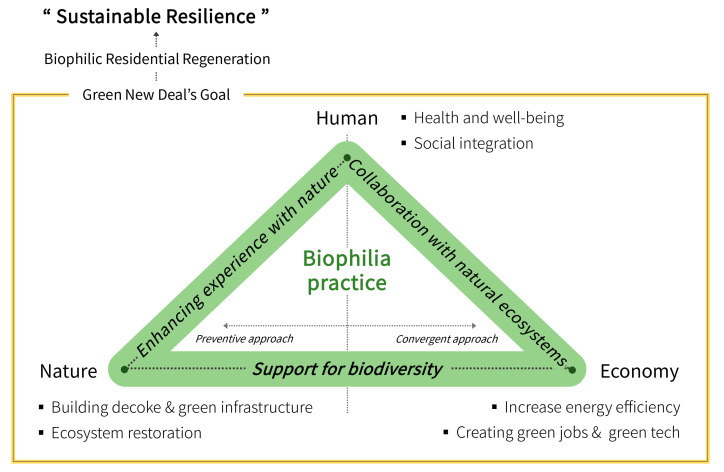
The link between the Green New Deal and Biophilia.

Biophilia refers to the instinctive love for life and natural systems and promotes the restoration of nature and human relations based on human emotional partnerships in natural life [10]. Edward O. Wilson, who popularized the biophilia hypothesis, suggests that the most serious disaster of climate change is the loss of genetic diversity due to natural habitat destruction [38]. This is because being alive and staying healthy when activating the love of life within [39], especially exploring and feeling close to life, is an essential and positive process for mental health [10]. In other words, humans and society cannot be completely healthy in a modern urban environment that lacks access to nature and should provide a more renewable and resilient environment through the restoration of natural and human relations. Resilience is the ability of a system to derive profits by absorbing and utilizing psychological disturbances and changes and has a sustainable structure [40]. The resilience of a city based on biophilia requires a shift in the idea of recognizing natural resources and biodiversity as the basis of support for survival, and it is important to strengthen local residents’ instinctive attachment to nature. In this context, this study focused on the similarity between the Green New Deal goals and the biophilia hypothesis, seeking benefits through positive ties between humans and nature, and drew a theoretical basis for applying and integrating residential regeneration. Figure 2 shows its link with the biophilia hypothesis from the Green New Deal perspective.

The Green New Deal is being discussed in terms of humanity, nature, and economy, and this study derived three concepts for the practice of biophilia from the perspective of the Green New Deal. The first is “support for biodiversity”. The clearest common thing in the practice of the Green New Deal and biophilia is the preservation of the ecosystem, specifically an active attitude toward securing various species. Securing biodiversity supports diverse experiences with nature and contributes to enhancing the competitiveness of the community by assuring the evolution and specificity of indigenous creatures. In other words, biodiversity based on bioethics serves as an organism of a complete ecosystem by inducing continuous well-being of the individual and economic society of the city. The second concept is “enhancing experience with nature,” which is to improve the bond between humans and nature through human physical, physiological, and mental characteristics that respond positively to nature and further ensure the health and healing of local residents. Prior studies related to human responses to nature have provided convincing evidence in a wide range of fields in which interaction with nature is related to inducing healing and recovery, improved understanding of learning, and reduced social hostility and aggression [13,17,41,42,43]. Recently, the architectural community has discussed planning and technical measures to maximize the experience with nature to build a healthy physical environment and a high-quality living environment through the effects of biophilia [44]. The third and last concept is the “collaboration with natural ecosystem”, which is to recognize that all-natural resources are finite and pursue a cooperative relationship between humans and nature rather than a one-sided collection of resources. This needs to be considered in the Green New Deal plan, regarding the concept of biomimicry [11], which derives innovative ideas by imitating the principles of biology and creating new technologies and values in implementing eco-friendly buildings. The approach from collection to collaboration has a structure in which mutual benefits are generated in the process of utilizing resources, and this mutual benefit further strengthens access and relationship to nature.

From the perspective of the Green New Deal, biophilia provides interaction-based benefits through a complex process and ultimately ensures a healthy life for local residents, which can give new value to the existing Green New Deal strategy. Therefore, it is necessary to discuss specific linkages when building an urban and residential environment.

### 2.3. Residential Regeneration and Biophilic Design

Individual emotions and perceptions related to environmental preference are based on the characteristics of places where people can view the landscape and discover resources or hide themselves from dangers or threats [45,46,47]. The human mind, structure, and capacity, were formed through the process of living in the natural world, and its senses and tendencies developed according to the environmental characteristics of nature and way of life [48]. In just a few decades, the population of cities around the world has soared, leading to continued construction processes focused on efficiency and convenience, leaving humanity far from nature. Considering the history of lifestyles from an evolutionary perspective, biologically, there is not enough time for humans to fully adapt to a city. In this context, a plan to build an environment to promote and strengthen human interaction with nature was required. In architecture, biophilic design is being explored as a strategy to interpret biophilia and introduce it to the architectural environment. Eco-friendly and sustainable architecture and, recently, resilient living environments are discussed as major planning techniques [49,50,51].

Beatley [52] developed the “Nature Pyramid” as a metaphorical tool for suggesting nature’s recommendation and diversity of experience depending on the scale of the environment. It emphasizes that “everyday neighborhood nature” is very important in conveying the essence of a happy and healthy life. Although international and national-scale Mother Nature, at the top of the pyramid, guarantees a high level of values and rewards, it does not guarantee equal access; it is often difficult to experience and cannot be included in a natural diet of all scales. In other words, the application of the biophilic design should take into account contact with the nature around us, and it is important to restore the ecosystem on the residential scale and secure the biodiversity in the local environment. For example, if multi-family housing or apartment types are generalized depending on the population distribution, geographical characteristics, and scale limitations of each region, the experience with nature can be further weakened, so a detailed plan according to the scale of the residential area is required.

This study regards residential regeneration considering biophilic design for residential environments that are closely connected to daily life, and details on the scale and scope of residential regeneration are as shown in Table 1.

As mentioned previously, it is important to understand the role and potential value of biophilic design from the perspective of the Green New Deal to regenerate residential areas responding to climate change and urban problems. This study analyzed the expected effects of biophilic design considering the Green New Deal and the benefits of biophilic design for residential regeneration. The benefits were summarized in view biophilic characteristics and biophilia practice through consideration of the literature and prior research related to biophilic design. Table 2 shows the characteristics and benefits for the expected effects of biophilic design.

The biophilic design has expected health, economic, and social effects, and offers a wide range of benefits through direct and indirect contact with nature, a level of awareness of biophilia, and interactions with animals and plants. The design method of promoting experience with nature should support active physical activities and healthy mental conditions and create job positions for securing natural resources and managing the ecosystem. It should also respond to the vulnerability of the community, reducing hostility and aggression by promoting a friendly atmosphere and social interaction. In particular, the development of smart technology related to the cultivation of urban agriculture and microorganisms expands the residents’ understanding about biophilia and contributes to enhancing biophilic tendencies. The specific benefits of biophilic design for health contributes to the well-being of local residents and public health and provides economic and social benefits through biologically positive responses. This leads to the stabilization of economic and social structures, thereby maintaining the quality living and contributing to the formation of a healthy and resilient community.

## 3. The Biophilic Design for Residential Regeneration

### 3.1. Methodology of Study

This study analyzed literature and preceding research on biophilic design in a wide range of areas, including urban planning and residential regeneration, architectural planning and design, housing technology, and residential services to identify the features of biophilic design for the residential regeneration in various aspects. Table 3 summarizes the keywords used in this study.

**Figure 3:**
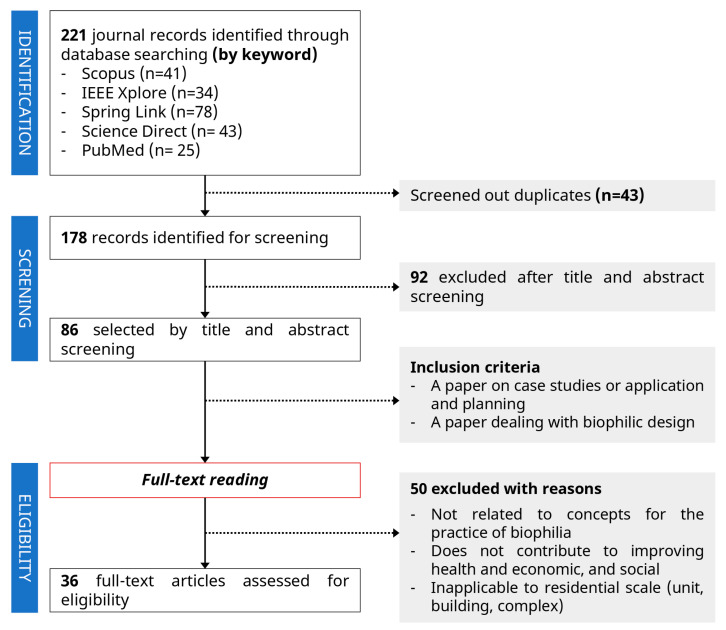
Literature review process.

The information was collected through major academic databases, and the collected data examined the relevance and applicability by referring to the biophilic design elements and patterns presented in the referenced study. Figure 3 shows the literature review process.

This study prioritized whether the biophilic characteristics and cases proposed by literature and articles are related to the three biophilia practice concepts. In addition, we valued the characteristics and cases that can provide health, economic, and social effects from the perspective of the Green New Deal and reviewed the applicability and application plan considering the scale of residential regeneration proposed in this study. The literature and articles collected through keyword searches were first selected through title and abstract screening after excluding redundancy; the final 36 papers were selected according to the analysis criteria after full-text review.

### 3.2. Results of Biophilic Design Features

This study analyzed the main characteristics and related examples of the application of biophilic design presented in the literature and previous studies according to the concept of three biophilia practices considering the Green New Deal. The biophilic feature considers the characteristics of planning elements and case objects that are common in literature, and includes five for support for biodiversity, five for enhancement of the experience with nature, and two for collaboration with natural ecosystems.

As a result of the literature review, the application of biophilic design in the field of planning and design varies depending on the use and size of a building and the perspective of the researcher. In particular, policy efforts depending on the size of the city are being made to preserve or utilize the environmental characteristics of the region, such as mountainous and coastal areas, but there is a lack of research on specific criteria for the application of biophilic design. In addition, the application of biophilic design for housing and residential areas is insufficient due to the concentration of public facilities and ecological tourism. In the field of technology and services, the development of new technologies has improved the availability of biophilic design. However, the development of immersive technology centered on the games, and entertainment, and the smart home industry focused on energy efficiency and sustainability needs to be directed to a more positive experience of humans and nature.

#### 3.2.1. Support for Biodiversity

The support for biodiversity guarantees and manages the right to survive for all species, creating an ecosystem of cities where both terrestrial and aquatic species coexist. Table 4 shows the literature and prior research analysis related to support for biodiversity.

The continuous landscape of the entire habitat through green rooftops and vertical gardens helps the dispersal of animals and plants. Securing native species in the residential environment encourages local residents to bond and creates new jobs to preserve the ecosystem. In addition, small ecosystems help reducing maintenance costs in residential complexes by forming circular structures, ultimately contributing to securing regional competitiveness.

#### 3.2.2. Enhancing Experience with Nature

Enhancing experience with nature is about creating opportunities and inducing contact with nature in a variety of ways. Nature is divided into direct, indirect, and vicarious (symbolic nature), according to the type of contact [91]. Direct nature is in direct contact with the natural environment, and the more you touch, feel, and look, the higher its value. Indirect nature is a controlled environment that can be experienced in aquariums and museums and is in contact with processed nature, such as indoor gardens and botanical gardens. Finally, vicarious nature is a scene that expresses or describes nature, and is expressed in a realistic or metaphorical way, depending on the situation. Currently, vicarious nature includes smart-home technology for experiencing multisensory nature or immersive technologies such as VR (virtual reality) and AR (augmented reality). Table 5 shows the literature and prior research analysis related to enhancing experience with nature.

The greening of community space for enhancing experience with nature enhances access to nature and affects social friendship and integration, responding to social vulnerabilities, such as reduced crime rates, increased consideration, and altruistic behavior. In particular, the link between biophilic design and smart-home technology can maximize direct contact with natural factors, such as daylight, wind, and climate change, and if it actively utilizes technologies that promote experience with nature inside [44], it can ease physical space constraints and improve the living environment and the quality of health and welfare of residents.

#### 3.2.3. Collaboration with Natural Ecosystems

Collaboration with natural ecosystems focuses on the symbiotic relationship between humans and nature rather than the collection of resources [73,99], promoting a mutual benefit from the development of architecture and technology. The development of sectors and technologies in the industries involved has unlimited potential and can lead to innovations in technology and profits to the community. Inspired by biological systems, collaborating with various species benefits both corporate and local ecosystems and residents, and it is an effective way to meet global needs. Table 6 shows the literature and prior research analysis related to collaboration with natural ecosystems.

Examples showing collaboration with natural ecosystems are applied to a wide range of areas ranging from environmental planning and design of architecture to technology and services; the tendency of biophilia can be strengthened by helping local residents understand and feel nature and biological systems, as they are closely related to their daily lives.

## 4. Biophilic Residential Regeneration for the Green New Deal

### 4.1. Integration of Residential Regeneration and Biophilic Features

**Figure 4:**
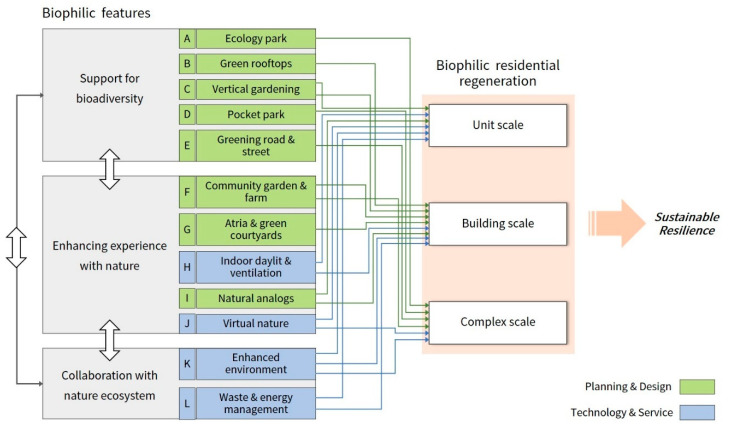
Integration pathways of biophilic design features considering the scale of residential regeneration.

This study derived a total of 12 biophilic features for residential regeneration and was divided into features of planning and design and features of technology and services, based on a review of the literature. The biophilic features of planning and design include elements and expression techniques of biophilic architecture, such as green rooftops, vertical greening, and natural analogs. The features of technologies and services include technologies of immersive experiences that support experiences and relationships with nature, such as virtual nature, improved environment, and waste and energy management, and elements of smart-home services. Based on the analysis, this study proposes a path to integrate the biophilic design and regeneration of residential areas; details are as shown in Figure 4.

This study classifies biophilic features according to the concept of biophilia practice; however, each feature is closely related to each other and forms a sustainable and resilient structure through a complementary relationship. Restoring wild nature and inducing life’s habitats around us to support biodiversity leads to opportunities for contact with nature in our daily lives, strengthening a positive natural experience. Activities to strengthen experience with nature promote biodiversity and require collaboration with the natural ecosystems for sustainable experiences. In addition, being inspired by nature or collaborating with the natural ecosystem is based on biodiversity, which requires a balanced approach to the three concepts of practice, and it is important to lay the foundation by applying it to a small environment first.

### 4.2. Strategies of Biophilic Residential Regeneration

Based on the literature and case studies, this study explored the application of biophilic features (A-L) considering the scale of residential regeneration, through which we propose a biophilic residential regeneration strategy. Table 7 shows the biophilic residential regeneration strategy and its associated benefits.

The unit scale is important to maximize direct and indirect contact with nature in limited indoor spaces. This requires housing technologies and services that can sense and respond organically to external natural environments. In addition, it is necessary to induce a visual or tactile connection with the indirect nature by utilizing the colors and materials of interior elements such as walls, ceilings, and floors. With the recent development of immersion technology, it is possible to simulate natural environments [44], and the construction of a natural immersive environment in a house using this innovation can be an original means to improve access to nature. The unit-scale biophilic strategy actively contributes to the improvement of residents’ health and well-being and helps reinforce the residents’ biophilia propensity to nature.

Biophilic strategies for building scale can support a continuous landscape outside and inside a building, while enhancing the functionality of the building and communication among residents. It is important to tend to indigenous species through rooftop landscaping to form small community farms and establish a self-sustaining system of buildings through natural resources and microbial culture environments. This increases the efficiency of building energy and space promotes social exchange and contributes to securing food resources.

Biophilic strategies for the complex scale is a strategy for residential activation and community awareness, including residents’ participation in managing ecosystems near residential areas. The extensive greening of the complex, such as pedestrian paths, and street and pocket parks, improves water treatment and circulation systems and has a positive effect on the improvement of the microclimate. In addition, public design and community parks in residential complexes that take into account biodiversity protection and virtual nature contribute to building biological knowledge, strengthening community awareness, and revitalizing residential areas. Biophilic residential complexes support the sustainability of biophilic units and buildings, while also providing the foundation for biophilic cities.

## 5. Discussion

The healthy environment required by the Green New Deal can be strengthened by biological characteristics based on the relationship between humans and nature and, ultimately, contribute to the sustainable health and well-being of local residents. Prior research on biophilic design has played a significant role in shaping the biophilic system, emphasizing the role of the architectural environment for health and well-being. However, we often tend to overlook everyday places in the urban environment; in particular, there should be sufficient discussion about the minimum natural level or approach for local residents and neighbors. There is also a lack of consideration for technical and smart methodologies to enhance bio-friendliness. Therefore, from a multifaceted and long-term perspective, the biological investment for public health of the community is a very important research topic. This study deals with how to respond to climate change and urban vulnerabilities and apply biophilic design as a strategy for residential regeneration for quality residential life.

Interaction of people with nature promotes the recovery and well-being, acting as a preventive factor related to everyday stress. This can promote climate stabilization and regional competitiveness by driving meaningful interactions for the health and sustainability of communities. It also contributes to public health by ensuring equal access of residents to nature. Therefore, the government-led Green New Deal project needs a residential regeneration strategy that strengthens the organic linkage of biophilic design and maximize the effectiveness of financial expenditure in the long term.

To support biodiversity in continuous landscaping of various sizes, from indoors to buildings to residential complexes and cities, all creatures should coexist through strategies tailored to the biogeographic characteristics of the region. The experience with nature should be discussed in various aspects, and technical services should be provided in the scale of the unit and building for the equality of natural contact. In this process, companies can not only obtain innovative ideas but also have to develop sustainable products and processes, which would serve as an opportunity to respond to external and internal environmental vulnerabilities.

Finally, the concept of biophilia practice proposed in this study is a complementary relationship, taking into account the characteristics, housing type, and scale of each local environment to secure biodiversity, strengthen experience with nature, and facilitate collaboration with the natural ecosystems. In unit scale, it is necessary to utilize various technologies to bring the external natural environment indoors and to develop the contents of the user’s experience. Moreover, considering the improvement of the health and energy efficiency of residents can be a cost-effective alternative in the long term. Building scale shall provide residents with experiential opportunities of nature through rooftops and public spaces and shall establish a system of buildings to support the cultivation of microorganisms and the utilization of natural resources. This contributes to the provision of a certain amount of healthy food and to the improvement of building efficiency. On a complex scale, it is important to create small green spaces and a bio-friendly ecosystem. Services and contents indicating indigenous plant species and surrounding environmental characteristics in the residential complexes continuously maintain and promote bio-friendly units and buildings and contribute to the improvement of the residents’ community awareness.

## 6. Conclusions

A key premise of this study is that there is a close link between biophilia or biophilic residential regeneration and recovery-resilient communities and healthy residents, where specifically, the former helps develop the latter. The extent to which local residents can be termed ‘bio-friendly’ depends on their preference for the natural environment around them, the level of participation in their experience with nature, or the social attitude that supports them. In other words, the biophilic residential environment should help residents actively interact with nature, enjoy nature, and take care of it. However, more efforts and research are needed in the future for biophilic residential regeneration. For example, short-term economic costs for green roofs and building systems can hinder the creation of a bio-friendly environment. Whilst implementation of these strategies requires policy investment and management, it also leads to long-term cost savings. In addition, to provide biophilic services, it is necessary to provide indicators to understand and measure specific environmental factors favored by local residents, as well as change the modern lifestyle that relies on the means of living indoors and transportation.

The study emphasizes that the Green New Deal project should focus on linking essential relationships rather than on the superficial mutual development of human, natural, and economic benefits. In addition, various methods to apply a bio-friendly design in residential regeneration were explored. The biophilic residential regeneration proposed in this study is worthy of use as a series of indicators that contribute to the Green New Deal and the resilience of the community. This contributes to expanding the understanding of biophilic design and residential regeneration from the perspective of the Green New Deal and provides insight into the conversion of biological ideas into the market in architectural planning and technology development. This study deals with the developed domain of existing theories about the topic of the study and has a challenging value in that it takes into account the linkage with other related variables.

However, by utilizing the few categories presented in the literature and previous studies, this study is limited in comparing and analyzing specific applications, taking into account the characteristics of the local environment and residents and in buildings on clear criteria for the application of biophilic design. This is because the concept of biophilic design is often ambiguous and hardly defined as a single analytical standard, as it is diversified and complex based on the respective researcher and the field and scale of the application. Nevertheless, this study proposes a meaningful theoretical basis for integrating the concepts of the Green New Deal and biophilia, and proposes a detailed plan considering the scale of residential regeneration, along with the features of biophilia based on the concept of biophilia practice. In future research, it is important to establish a system of guidelines that can ease constraints and limitations in the use of biophilic residential regeneration strategies. It is further necessary to specify a strategy for biophilic residential regeneration by considering the cultural characteristics of local residents, the environmental features of local ecosystems, and the level of technology.

## Figures and Tables

**Table 1:** Range of residential regeneration.

Scale	Range
Unit	Unit space in a house, including interior environmental planning (living room, bedroom, kitchen, etc.) and intelligent residential services
Building	A plan for energy management and shared space (piloti, lobby, rooftop garden, etc.) considering multi-family housing and apartment types
Complex	A plan for joint facilities (parking lots, parks, etc.) and access road & street for residents of nearby houses (building)

**Table 2:** Characteristics and benefits of the biophilic design.

Expected Effect		Biophilic Design Characteristics	Biophilia Practice	Benefits	Resource
Healthy	Physical	A walk in the park and woods	Ee	Improved walking and balance; reduced falls	[53,54]
		Natural material; green wall	Ee	Reduced respiratory diseases; relieved headaches and dizziness	[55,56]
	Physiological	A walk in the park and woods; visual exposure to nature	Ee	Reduced blood pressure and heart rate; decreased sympathetic nervous system activity	[41,57,58]
		Plant cultivation; gardening activities; indoor garden	Ee	Promoted neuroendocrine system and stress recovery	[11,59]
		Daylight	Ee	Improved life cycle and biorhythm	[60,61]
		Green space close to homes	Sb	Improved average life span	[62]
	Psychological	Visual exposure to nature	Ee	Improved subjective satisfaction; reduced anxiety and tension, frustration, boredom, and fatigue	[63,64]
		A walk in the park and woods	Ee	Improved observation, attention-inducing, concentration, and problem-solving skills	[53,65,66]
		Natural color and pattern; Nature sound	Ee	Improved creativity and emotional recovery; reduced loneliness	[67,68,69]
Economical	Healthcare savings	Biodiversity	Sb	Decreased disease rate and number of visits to medical institutions	[8,64]
		Visual exposure to nature	Ee	Decreased hospitalization and pain medication doses	[48,70]
		Daylight	Ee	Reduced sick leave and absence of students and workers	[71,72]
	Reduced energy consumption & maintenance costs	Green building system; green wall	Cn	Increased natural purification; reduced building maintenance costs	[14]
		Rainwater recycling and solar heat energy	Cn	Secured renewable energy; reduced energy consumption	[14,44]
		Microbial culture; community farm	Cn; Sb	Reduced waste disposal costs; secured food resources	[73,74]
	Revitalizing local economy	Urban forest restoration; forestry; urban agriculture	Cn; Ee	Created jobs for local residents; secured regional competitiveness	[74]
		Water space; daylight	Ee; Cn	Increased local store sales and real estate value	[14,75]
Social	Enhancing community	Green space close to homes	Sb	Increased frequency of use of public spaces and regional community	[76,77]
		Gardening activities; foodscaping; animals	Ee	Improved conversations, affinities and preferences	[78,79]
	Urban vulnerability reduction	A walk in the park and woods; nature sound	Ee	Reduced crime rate, aggressiveness, violence, and fear	[17,77,80]
		Roadside trees; vertical greening	Sb	Reduced traffic accidents; revitalized communities	[51]

Note: Sb = Support for biodiversity, Ee = Enhancing experience with nature, Cn = Collaboration with natural ecosystems.

**Table 3:** Keyword for literature review.

Keyword & Criteria	Contents	
Keyword	City and urban planning	“Biophilic city”, “Biophilic urbanism”, “Urban greening”, “Urban generation”
	Residential regeneration	“Biophilic residence”, “Biophilic residential environment”, “Biophilic community”, “Biophilic living”, “Residential generation”
	Design of architecture and space	“Biophilic design”, “Nature-based design”, “Biophilic architecture”, “Biophilic building”, “Biophilic indoor environment”
	Technology and service	“Biophilic technology”, “Sustainable technology”, Biophilic service”, “Biophilic design industrial”, “Nature immersive”, “Smart-home technology”, “Smart-home service”

**Table 4:** Biophilic features related to support for biodiversity.

Biophilic Feature		Biophilic Examples		Resource
A	Ecology park	Nature-Park Sudgelande, Berlin, Germany (2000)	Creation of a nature reserve through the participation of local residents to preserve the biodiversity around the closed Sudgelande Railway; used as parks and exhibition spaces for local residents and artists	[13,74,81,82]
		Greenways for Pittsburgh program, USA (1980)	Open space for excellent management and conservation of ecology; creation of walkways and bike paths for local residents; creation of local jobs for environmental monitoring and maintenance	
B	Green rooftops	Podium Green Roof, Toronto, Canada (2009)	Utilization of modular systems comprised of indigenous species; plant species placement taking into account site conditions such as daylight and shade; habitat provision for insects and birds	[74,83,84,85,86]
C	Vertical gardening	Optima Camelview Village, Arizona, USA (2007)	Townhouses; creation of a variety of vegetation and green spaces on bridges and balconies connecting buildings; the provision of a series of landscapes throughout the complex	[13,15,51,83,86,87]
		Bosco Verticale, Italy (2014)	Residential towers; provision of tree planted on balconies in each unit space; arrangement of tree species according to the height and direction of each building	
		Façade of Quai Branly Museum, France (2006)	Support for the reproduction of various organisms, such as moss and insects; used materials such as green wall façades, stones, and trees	
D	Pocket park	Parklet, California, USA (2005)	Green spaces and pocket parks using parking spaces in creative ways for each residential complex; access to residential complexes and roads through green areas	[16]
E	Greening road & street	Green street program Portland, USA (2007)	Management of rainfall runoff through public facilities including planting; prevention of illegal parking through planting expansion facilities on roads and street pots; increase of superior infiltration surface	[13,16,74,84,88,89,90]
		Wildlife passages, Canada (2007)	Creation of movement routes for animals, avoiding roads to connect animal habitats; construction around the upper part of tunnels, underground parking lots, and roads to protect protection local animals and ecosystems	

**Table 5:** Biophilic features related to enhancing experience with nature.

Biophilic Feature		Biophilic Examples		Resource
F	Community garden & farm	Paley Park, New York, USA (1967)	A small park between buildings in the city; an ivy, a tree canopy, and artificial waterfalls to increase the sounds of nature and reduce the noises in the city	[16,48,92,93]
		Phipps Conservatory, Pittsburgh, USA (1893)	It plays the natural recorded sound of Pittsburgh through 12 speakers in the main atrium; autoplay based on seasonal and climate conditions	
		Via Verde, NewYork, USA (2012)	Multi-family housing; terraced residential complexes to create walkways by height and communal gardens and to induce exploring experiences of vicarious nature	
G	Atria & green courtyards	Khoo Teck Puat Hospital, Singapore (2010)	An indoor garden with waterfalls; a city farm on the roof of a building; provision of a shared space for local communities	[13,16,94,95]
		Victoria Park Villas, Singapore (2018)	A courtyard that can be viewed from any unit; the provision of natural light and airflow in the interior through skylight and louver; the provision of a transparent window for the privacy and view of the courtyard	
H	Indoor daylit & ventilation	One Central Park, Australia, Sydney (2013)	Residential towers; secured natural lighting of shaded areas through heliostats installed between two buildings	[16,44,96]
		LS skylights; HC moons (CeoLux)	Artificial lighting display systems; the use of LED technology to recreate the spectrum of real sunlight and moonlight; virtual appreciation of the sky and climate changes	
		Prolouver (Pergola)	Environmental sensor + controller + actuator coupling unit; provision of an optimized indoor environment, according to rainwater, light, and wind direction; provision of comfort and stability from the natural environment	
I	Natural analogs	Ekouin Nenbutsudo Temple, Japan (2013)	The use of natural materials such as trees and stones in every space; the use of colors and patterns to symbolize nature	[13,97]
		Aqua Tower, Chicago, USA (2007)	An organic building in the shape of waves; prevention of collisions between buildings and birds through the shape of glass finishes and curves	
J	Virtual nature	Maplewood Senior Living, Westport, USA (2017)	Provision of “sky lounge” with immersive displays and audio, videos, and images of nature; support of real-time communication with friends and family	[44,96,98]
		Undersea Project (Magic Leap)	Implementation of elements of nature’s environment through technologies of augmented reality and mixed reality; support for experiences with living things based on virtual natural environments (dolphins, birds, etc.)	
		Komorebi (LESLIE NOOTEBOOM)	A project lamp that generates shapes and shadows of virtual light; provision of the properties of light reflected in water or reflected between leaves	

**Table 6:** Biophilic features related to collaboration with natural ecosystems.

Biophilic Feature		Biophilic Examples		Resource
K	Enhanced environment	Supertree Grove in Gardens by the Bay, Singapore (2012)	Public structures with technologies that mimic the ecological functions of trees; shades, solar energy, and rainwater recycling; provision of habitats for insects and birds	[88,100,101,102]
		Nedlaw Living Walls (Biofilter company)	Provision of indoor air quality and humidity control through wall recording; reduction of >90% of energy compared to existing HVAC ^1^ systems; provision of optimal plant cultivation conditions using automatic irrigation systems	
		Smart Aquarium; Smart Plants Growers (Multiple companies)	Devices for fishery harbor and plant management using the Internet of things; creation of an optimal growth environment with minimal management; provision of living conditions and related information; contribution to strengthening biophilia propensity	
		Termite Humidity Damping Device (Terrapin Bright Green)	Passive humidity damping device based on fungal combs in termite mounds; stabilization of humidity in buildings and reduction of energy demand	
L	Waste and energy management	Microbial Home (Phillips design, 2011)	Generation of bacteria and biogas energy through the decomposition of food and plastic waste; biogas energy utilization for cooking or cultivation of food, indoor lighting, etc.	[73,103,104,105]
		Aquaponic Systems (Multiple companies)	Hydroponics and fish farming based on symbiotic relationships between fish and plants; fish waste provides nutrients to plants, who filter the water for fish	
		Biolytix (Biolytix)	Household wastewater treatment systems that rely on worms and other organisms to filter water and break down sewage; exclusion of toxic chemicals; 90% energy saving compared with conventional sewage treatment system	
		Pilus Cell (Pilus Energy)	As modified bacteria break down organics in wastewater, producing electricity, treated water, and useful chemical compounds	
		Latro Lamp (Mike Thompson)	A lighting device that extracts electricity during photosynthesis of the lamp’s algae through sunlight, CO_2_, and water	

Note: ^1^ HVAC: heating, ventilating, and air conditioning.

**Table 7:** Biophilic residential regeneration strategy and benefits.

Scales	Biophilic Features	Strategies	Specific Benefits	Common Benefits
Unit	C	Balconies of each unit of housing for vegetation	Biodiversity; enhancing access to nature	Improved health and well-being Reduced building energy demand Reduced illness Increased productivity Increased property value Increased food security Extended infrastructure longevity Reduced UHI ^1^ Improved air quality Enhanced water quality(filtration) Residential regeneration Encouragement physical activity Provided recreation Increased regional competitiveness Increased community sense
		Bio wall (green wall) with built-in automatic irrigation system	Improved indoor air quality	
	H	Automatic actuator (louver; curtain; window; etc.) according to daylighting and wind direction	Improved indoor air quality; maximization of daylight;	
	I	Interior design using natural shapes and patterns	Reduced stress; emotional stability	
		Using of natural colors and materials	Reduced stress; emotional stability	
	J	Smart-home device that provides virtual nature (3D object; video; etc.)	Enhancing access to nature; reduced stress	
	K	Smart aquarium; smart plants growers	Enhancing access to organism; reduced stress	
	L	Waste treatment system through microbial culture	Reduced waste disposal costs	
Building	B; F	Green rooftops and foodscaping considering indigenous species	Improved space efficiency; securing food	
	C; K	Façade greening and vertical greening system using rainwater	Improved water management and energy efficiency	
	C	Green corridors between buildings	Improved visual amenity; biodiversity	
	G	Nature-friendly sharing space in atria and courtyards	Promotion of communication	
	G; H	Heliostate for solar path tracking and daylight streams	Maximization of daylight	
	I	Organic building shapes and forms for bird protection	Improved visual amenity; biodiversity	
	L	Water treatment and energy management system based on microorganisms	Reduced waste disposal costs; improved energy efficiency	
Complex	A	Linkage between ecological parks and residential complexes; participation of residents for ecological management and operation	Promotion of communication; creates employment	
	D	Parklet utilizing parking space	Biodiversity; improved visual amenity	
	E	Shade planting for buildings placed to remove heat load	Prevention of heat load	
		Green permeable sidewalks; plantation expansion facilities	Prevention of flooding	
	F	Community parks that include artificial waterfalls, fountains, and sounds of nature	Promotion of communication; enhancing access to nature	
	J; K	Public design considering the habitats of urban creatures and virtual nature	Biodiversity; enhancing access to nature	

Note: ^1^ UHI: Urban heat island.
